# Copper-selective electrochemical filling of macropore arrays for through-silicon via applications

**DOI:** 10.1186/1556-276X-7-375

**Published:** 2012-07-09

**Authors:** Thomas Defforge, Jérôme Billoué, Marianne Diatta, François Tran-Van, Gaël Gautier

**Affiliations:** 1 Université François Rabelais de Tours, GREMAN UMR CNRS 7347, 16 Rue Pierre et Marie Curie, BP 7155, Tours Cedex 2, 37071, France; 2 Université François Rabelais de Tours, Laboratoire de Physico-Chimie des Matériaux et des Electrolytes pour l'Energie, E.A. 6299, Parc de Grandmont, Tours, 37200, France

**Keywords:** Silicon electrochemical etching, Macropore arrays, Copper electrochemical deposition, Through-silicon via

## Abstract

In this article, the physico-chemical and electrochemical conditions of through-silicon via formation were studied. First, macropore arrays were etched through a low doped n-type silicon wafer by anodization under illumination into a hydrofluoric acid-based electrolyte. After electrochemical etching, ‘almost’ through-silicon macropores were locally opened by a backside photolithographic process followed by anisotropic etching. The 450 × 450-μm² opened areas were then selectively filled with copper by a potentiostatic electrochemical deposition. Using this process, high density conductive via (4.5 × 10^5^ cm^−^²) was carried out. The conductive paths were then electrically characterized, and a resistance equal to 32 mΩ/copper-filled macropore was determined.

## Background

Nowadays, the scaling down (known as ‘More Moore’) of devices is coming to an end. To maintain a constant evolution of the device performances in the future, alternative ways must be explored. The main one is the device functional diversification and the three-dimensional (3D) integration (‘More than Moore’). The 3D integration is based on the use of the semiconductor volume to connect both sides of a silicon wafer [1,2]. This technique enables the stacking of the dies and leads to an important surface gain. The through-silicon via (TSV) technology is the key parameter for 3D integration allowing through-silicon connections. This technique is based on the etching of TSV and then the filling of these through holes by a conductive metallic material such as copper or tungsten [1].

The TSV are usually etched into the silicon using deep reactive ion etching (DRIE) [3,4]. This technique requires both chemical and physical silicon attacks [5] to achieve anisotropic etching of silicon. The Bosch process [6] is often employed to reach high aspect ratio (HAR) structures. It consists of the alternative insertion into the etching chamber of two gaseous species: an etching one (e.g., SF_6_) and a polymerizing one that immunizes the sidewalls from the SF_6_ attack. This enables the formation of versatile structures into the silicon wafer. Despite its high versatility, DRIE technique suffers of several limitations. The first limitation is the value of the via aspect ratio that can be attained. Indeed, this value is limited to around 30 or 40 because of homogeneity defects [7]. Furthermore, the main issue of DRIE is the corrugation (or scalloping) development on the silicon sidewalls. This corrugation is directly imputed to the alternative steps of the Bosch etching process. The scalloping is known to be responsible for deposition conformity defaults.

The electrochemical etching of silicon leading to porous silicon formation is an alternative to DRIE for low-cost TSV fabrication. Indeed, macropore formation (pore diameter > 50 nm and most of the time > 1 μm) presents several advantages as compared with dry etching technique. In this case, the wafers are immersed into a HF-based solution (often mixed with solvents and/or surfactants) and an anodic current (or potential) is applied to the sample. The morphology of porous silicon is influenced by the substrate nature (its orientation, doping type, and concentration), the electrolyte composition (HF and additive concentrations), and the anodization parameters (applied current or potential, illumination, etc.) [8]. To obtain HAR, high-density TSV, a low-doped (*ρ* > 0.5 Ω cm) n-type (100)-oriented substrate can be employed. For example, HAR macropores (aspect ratios can reach 250 to 300) [9] with ultra-high densities (>10^8^ pores/cm²) [10] can be achieved homogeneously on 6-in. wafers [11]. These values are much higher than those reached by DRIE and very promising for the future TSV density increasing needs.

Most of the time, TSV are filled with copper because this metal owns one of the highest electrical conductivity. Copper thin films are often obtained by electrochemical deposition. This technique is a low-cost way to achieve large area, high conductivity copper layers. Copper electrochemical deposition into through holes is thus studied for almost two decades because conformal deposition into confined media is much more difficult than on flat surfaces [12]. The chemistry of the electrolyte needs to be adapted to this constraint. Thus, several additives are commonly added to the H_2_SO_4_-CuSO_4_ electrolytic mixture during the copper deposition to ensure void-free, high conductivity copper filling even into HAR structures [13].

The first part of the present paper describes the different strategies to achieve conductive TSV from ordered macroporous silicon. Then, the conditions of HAR macropore array etching by silicon anodization were developed. After anodization, ‘almost’ through-silicon macropores were locally opened by backside photolithography. The through-silicon macropores were then filled with copper. Finally, these TSV were electrically characterized to determine the resistance of the conductive paths.

### Different strategies of conductive through-silicon via localization

**Figure 1:**
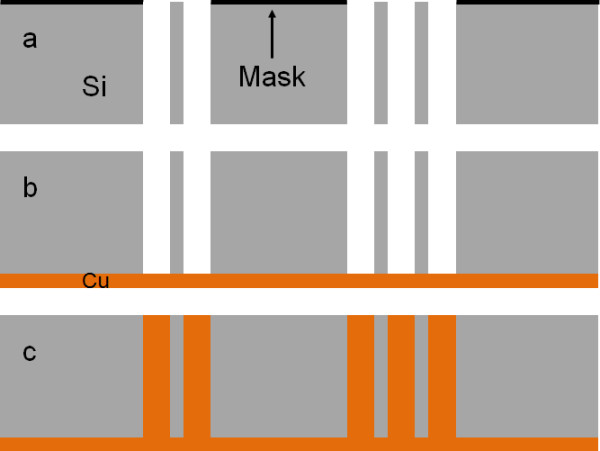
**Selective TSV formation by macropore array localization.** (**a**) Through-silicon macropores are locally etched by electrochemical etching through an inert mask. (**b**) The seed layer is deposited on the backside of the substrate. (**c**) The macropores are filled with copper by bottom-up electrochemical deposition.

To achieve localized TSV, four strategies can be explored. Either the macropores or the seed layer can be localized on the sample in order to select the conductive regions. The simplest way would be the ordered macropore array localization (such as DRIE technique) because this technique leads to the formation of local, through-silicon macropore regions depending on the mask design (*cf.*1a). After anodization, a simple backside seed layer deposited on the whole surface of the wafer ensures the metal electrochemical bottom-up filling (Figure 1b,c) [14]. However, this process is very difficult to perform because of macropore localization issues. Inert masking layers may be employed to protect some areas against the porous silicon formation, but over-etching is observed at the edge of unmasked regions [15,16].

**Figure 2:**
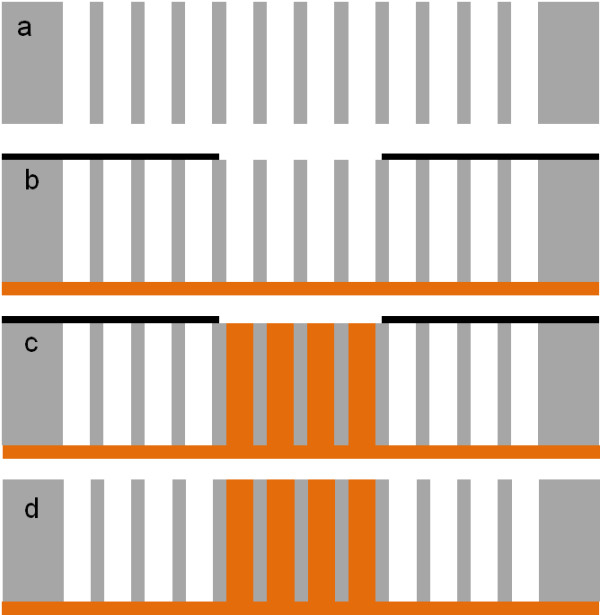
**Selective TSV formation by local macropore masking before copper electrochemical deposition.** (**a**) Electrochemical etching of silicon leading to through-silicon structures. (**b**) Local masking of macropores limiting the electrolyte penetration into opened regions. (**c**) Local filling of the macropore arrays with copper by electrochemical deposition. (**d**) Mask removal.

A second option could be the formation of macropore array on a whole sample and the localization of copper electrolyte penetration in the macropores by photolithography as described by Föll et al. [17]. Figure 2 illustrates the process flow. After the through-silicon macropore etching (Figure 2a), a masking layer is locally deposited on the pores to stop the penetration of the electrolyte (see Figure 2b). During the copper electrochemical deposition, the macropores in contact with the electrolyte are selectively filled with copper (Figure 2c). After the pore filling, the backside seed layer can be removed by a polishing technique (Figure 2d). The main issue of this process is the technique employed to locally mask a textured surface such as macropore arrays. It cannot be performed by usual photolithography.

**Figure 3:**
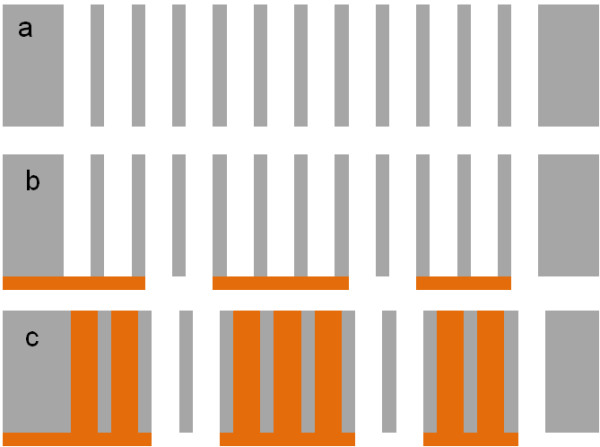
**Selective TSV formation by local seed layer deposition.** (**a**) Electrochemical etching of silicon leading to through-silicon macropore array formation. (**b**) Local deposition of the seed layer on one side of the sample. (**c**) The macropores sealed by the seed layer are selectively filled with copper.

The third strategy is the seed layer local deposition onto through-silicon macropores. After silicon anodization leading to through-silicon macropore formation (*cf.* Figure 3a), the seed layer is selectively deposited on the backside of the wafer (Figure 3b). For this purpose, ink-jet deposition technique as well as locally metalized contact wafer stacking can be employed. Since the copper can only grow on the conductive surface, the copper is limited to the seed layer areas (Figure 3c). Using this strategy, through-silicon via locally filled with copper may be obtained as already described in the literature [18].

**Figure 4:**
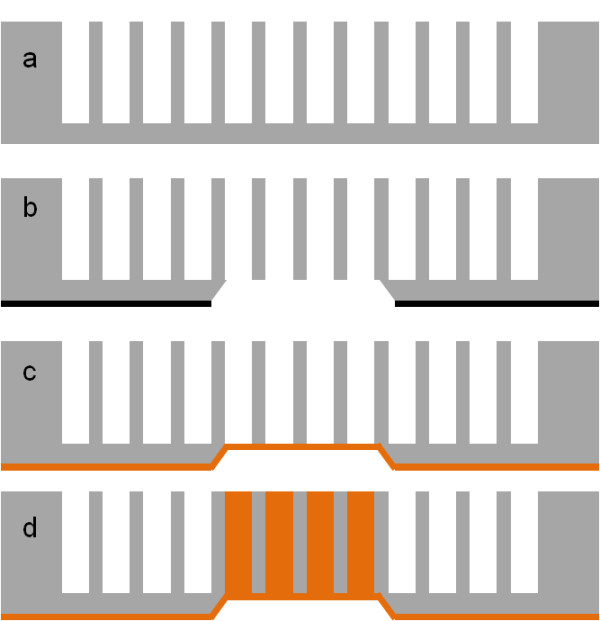
**Selective TSV formation by local opening of almost through-silicon macropores.** (**a**) Electrochemical etching of silicon leading to high aspect ratio structures. (**b**) Selective opening of the backside oxide mask followed by alkaline etching. This leads to local formation of through-silicon macropores. (**c**) Seed layer deposition on the backside. (**d**) Copper electrochemical deposition into the through-silicon macropores.

The last strategy is based on the local opening of almost through-silicon macropores [19]. Deep macropores were etched, but the anodization was stopped before the backside opening (see Figure 4a). After silicon dioxide (SiO_2_) thermal growth followed by backside photolithography, alkaline etching is performed to locally open the macropores (Figure 4b). The seed layer was deposited on the backside of the sample; thus, the filling of macropores is limited to ‘through’ regions (Figure 4c,d). This process was chosen to be tested because it can be performed using ordinary microelectronic tools. Finally, this process is much easier than direct macropore localization (strategy 1) because it limits the edge defects.

## Methods

### Macropore array electrochemical etching

To perform ordered macropore arrays, low phosphorus-doped (*ρ* = 26 to 33 Ω cm), (100)- oriented silicon was employed. The thickness of the wafers was 240 μm. Highly ordered macropore growth was ensured after inverted micro-pyramid array etching through an oxide mask using photolithography followed by alkaline etching (e.g., immersion into 20 wt.% of KOH solution at 80 °C until complete development of the pyramid). The samples were then anodized under galvanostatic control using a Bio-Logic® SP-150 (Bio-logic Science Instruments, Claix, France). During the anodization, the silicon samples were immersed into a HF (5 wt.%)-ethanol (20 wt.%) mixture maintained at ambient temperature. A 130-W backside illumination of the sample was also essential to photo-generate holes into the semiconductor. Anodization was stopped just before the backside opening; in the present case, approximately 220-μm deep macropores were etched (Figure 4a). The silicon electrochemical etching enabled the formation of 15-μm pitch, 12-μm diameter, with an aspect ratio of 18- to 20-almost-through macropore arrays. For this purpose, the current density was maintained equal to 14.5 mA cm^−^² during 4 h. This value ensures homogeneous macropore growth and porosity around 70 %.

### Copper electrochemical deposition

**Figure 5:**
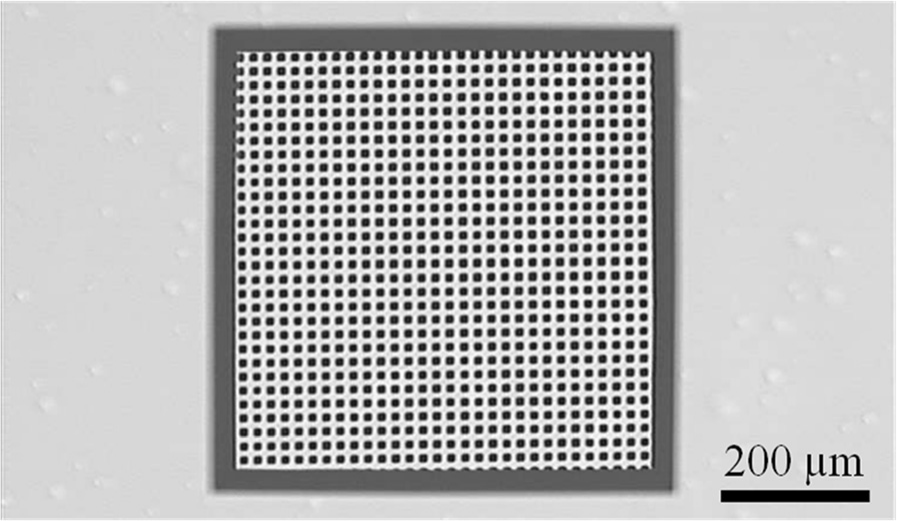
**Locally opened through-silicon macropores.** Optical microscope view of selective through-silicon macropore. This structure is achieved by alkaline etching (highly concentrated KOH solution at 80 °C) of the backside of the substrate through an oxide mask.

Once the macropores were etched, the sample was thermally oxidized. A photolithography step was performed on the backside of the samples to locally etch the oxide layer. The samples were immersed into the KOH solution. The oxide-free regions were etched until the macropore tips were attained (*cf.* Figure 4b). Actually, a thin SiO_2_ layer remained at the pore tips. In order to completely open the macropores, HF (50 wt.%) dipping was achieve until complete oxide removal. At this stage, 450 × 450-μm² areas were opened every 3 mm. Figure 5 illustrates one of these opened regions; every macropores were opened. Indeed, using this technique, local through-silicon macropores approximately 220-μm deep were formed. A second dry thermal oxidation was performed to isolate the silicon from the copper deposit and avoid parasitic copper growth as well as copper diffusion into silicon. Then, a non-conformal Ti/Ni/Au tri-layer was deposited by sputtering. This deposit ensures both good electrical contact and good adhesion on SiO_2_ (see Figure 4c). Moreover, the non-conformal deposition limits the next copper growth on one side of the sample (not on the sidewalls) for a bottom-up filling.

**Figure 6:**
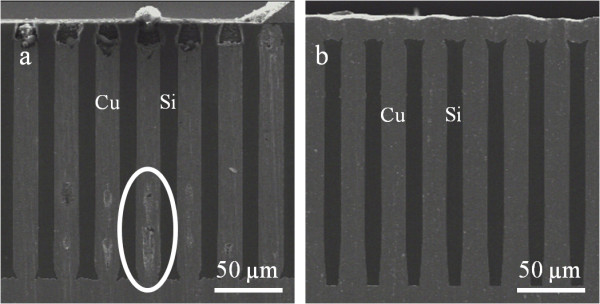
**Filling quality of the macropores with copper.** SEM polished, sliced views of copper-filled macropores comparing the filling quality of macropores with copper. (**a**) Using a non-accurate electrolytic solution (JGB concentration equal to 5 ppm) leads to void formation during the copper growth. (**b**) Using an adapted electrolyte (JGB concentration was increased to 10 ppm) leads to the formation of void-free copper deposition.

The macropore arrays were immersed into copper sulfate-sulfuric acid solution with 0.5 and 1 M as respective molar concentrations. H_2_SO_4_ and CuSO_4_ were also mixed with three additives. The chemical nature as well as the concentration of these additives is essential for an efficient pore filling. The accelerator enhances the deposition kinetic in confined medias (e.g., at the pore tip) [20]. It is always a sulfur-based molecule, and in the present study, a sodium sulfopropyldisulfite (SPS) was employed as an accelerator. The suppressor is a mixture of polyoxyethylene-based polymer (the most used being the polyethylene glycol (PEG)) and chloride ions (Cl^−^). This additive adsorbs at the surface of the sample and inhibits the deposition rate (i.e., it results in few tens of millivolt overpotential deposition) [21,22]. In this study, 1,200-g mol^−1^ polyoxyethylene lauryl ether (POE) was preferred to PEG, thanks to its higher inhibition behavior [23]. The last additive added to the electrolyte was the Janus Green B (JGB), a nitrogen-based molecule. It is known as a leveler; it limits mushroom-like copper overfilling at the end of the electrodeposition process. The presence of these three additives (SPS, POE-Cl^−^, and JGB) involves differential deposition kinetics between the seed layer and the surface. They limit the deposition outside of the seed layer and guarantee an important pore filling kinetic [24,25]. The polished, sliced scanning electron microscope (SEM) views in Figure 6 prove that the filling quality strongly depends on the electrolyte composition. In the first case (Figure 6a), the electrochemical deposition was performed into an electrolyte whose additive concentrations were not optimized (with 5 ppm, the JGB content was too low); voids developed during the electrochemical deposition step [26]. However, if the chemistry of the electrolyte is well adapted to HAR structures (the JGB concentration was increased to 10 ppm), no void was observed (Figure 6b). A copper overfilling is observed at the top of the structure (*cf.* Figure 6b). This surplus of copper was removed by polishing the top surface before the electrical characterization.

The copper filling was performed under potentiostatic control (−0.15 V vs. saturated calomel electrode reference) during 6 h to ensure a complete pore filling. The samples were then rinsed with deionized water, polished, and analyzed by SEM and optical microscope.

## Results and discussion

**Figure 7:**
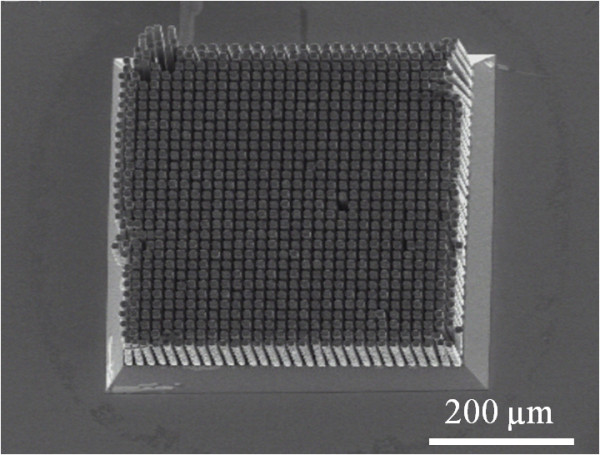
**Local formation of copper micropillar array.** SEM view of copper micropillar array formation after local copper growth into through-silicon macropore array followed by complete silicon removal by alkaline etching (KOH at high temperature).

To determine the copper growth homogeneity and the localization efficiency, silicon was selectively etched by a KOH solution at high temperature (80 °C). Thus, HAR copper micropillar arrays developed. Figure 7 illustrates one of these 450 × 450-μm² conductive paths. We can see a uniform copper growth inside the through-silicon macropore region. Outside this region, no parasitic copper growth was observed.

### Electrical characterization of TSV

**Figure 8:**
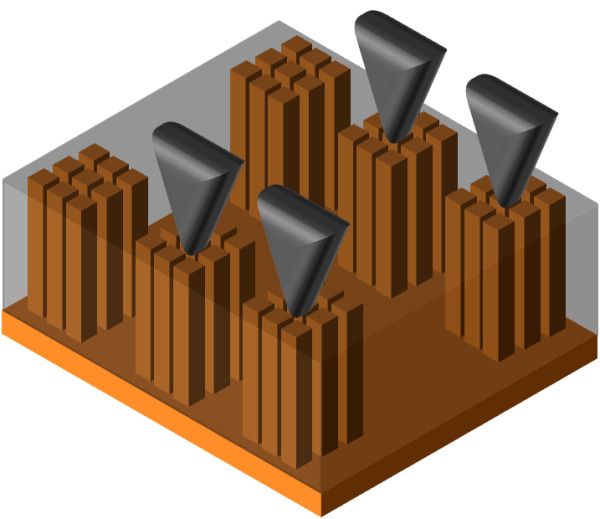
**Schematic representation of through-silicon macropore electrical characterization.** The four probes were apposed on the via and connected each other with the backside nucleation layer.

The conductive via grown into the macropore arrays were then electrically characterized. For this purpose, four-probe technique was employed. The probes were apposed on the copper-filled macropores and connected each other, thanks to the remaining backside nucleation layer (Figure 8). Voltamperometric scans were performed on a range of 0 to 100 mA to determine the resistance of the TSV. This resistance was found equal to 0.98 mΩ/32 via. After calculation, the resistance per via was found around 32 mΩ. The resistivity of the copper into the pores is approximately 1.78 μΩ cm; this value is 1.06 times higher than the theoretical copper resistivity (*ρ*_(Cu th.)_ = 1.67 μΩ cm) [27]. This measurement was performed several times to ensure a stable average resistance value. These results are better than those obtained in the literature for DRIE TSV [28]. The high copper conductivity into the pores may be explained by the slower deposition kinetic that limits void formation.

## Conclusion

After the determination of the different strategies to fabricate local TSV from macropore arrays, the selective opening of almost through-silicon macropores was chosen. Using a simple photolithography step, this technique enabled the local formation of highly conductive TSV. Macropores of 15-μm pitch and 12-μm wide were, thus, etched into a HF-based solution and then selectively filled with copper by a potentiostatic deposition technique during 6 h. During the deposition step, the electrolyte composition must be optimized to ensure void-free copper growth. Then, for the first time, the via resistivity was measured in a low-cost, full electrochemical (etching and filling) TSV fabrication process. The electrical characterizations demonstrated a low resistance of the conductive paths (32 mΩ/via). These values are coherent with the copper theoretical resistivity and explained by the slow copper growth into the macropores. This study proves the feasibility of TSV formation employing electrochemistry for the two main steps of the process (i.e., the pores etching and their filling with conductive material). Indeed, the ordered macropore electrochemical etching technique becomes a viable, low-cost alternative to DRIE for 3D integration.

## Abbreviations

3D, three dimensional; DRIE, deep reactive ion etching; HAR, high aspect ratio; JGB, Janus Green B; PEG, polyethylene glycol; POE, polyoxyethylene lauryl ether; SEM, scanning electron microscope; SiO2, silicon dioxide; SPS, sodium sulfopropyldisulfite; TSV, through-silicon via.

## Competing interests

The authors declare that they have no competing interests.

## Authors' contributions

TD wrote the manuscript and deposited the copper. TD and MD electrochemically etched the silicon substrate. JB performed the electrical characterizations of the TSV. FT-V and GG participated in the conception of the study and revised the manuscript. All authors read and approved the final manuscript.
